# Update on the General Practice Optimising Structured Monitoring to Improve Clinical Outcomes in Type 2 Diabetes (GP-OSMOTIC) trial: statistical analysis plan for a multi-centre randomised controlled trial

**DOI:** 10.1186/s13063-018-3126-1

**Published:** 2019-01-30

**Authors:** Sharmala Thuraisingam, Patty Chondros, Max Catchpool, Kim Dalziel, Jo-Anne Manski-Nankervis, Jane Speight, Elizabeth Holmes-Truscott, Ralph Audehm, Jason Chiang, Irene Blackberry, David O’Neal, Kamlesh Khunti, James Best, John Furler

**Affiliations:** 1 Department of General Practice, University of Melbourne, 200 Berkeley St, Carlton, VIC 3053 Australia; 2 Centre for Health Policy, University of Melbourne, Level 4, 207 Bouverie St, Carlton, VIC 3053 Australia; 3 School of Psychology, Deakin University, 1 Gheringhap St, Geelong, VIC 3220 Australia; 4 The Australian Centre for Behavioural Research in Diabetes, Diabetes Victoria, 570 Elizabeth St, Melbourne, VIC 3000 Australia; 5 John Richards Centre for Rural Ageing Research, Latrobe University, 133 McKoy St, West Wodonga, VIC 3689 Australia; 6 Diabetes Research Centre, University of Leicester, Gwendolen Rd, Leicester, LE1 7RH UK; 7 Lee Kong Chian School of Medicince, Nanyang Technological University, 50 Nanyang Ave, Singapore, 639798 Singapore

**Keywords:** Statistical analysis plan, Randomised controlled trial, Type 2 diabetes, Retrospective continuous glucose monitoring, General practice

## Abstract

**Background:**

General Practice Optimising Structured Monitoring to Improve Clinical Outcomes in Type 2 Diabetes (GP-OSMOTIC) is a multicentre, individually randomised controlled trial aiming to compare the use of intermittent retrospective continuous glucose monitoring (r-CGM) to usual care in patients with type 2 diabetes attending general practice. The study protocol was published in the British Medical Journal Open and described the principal features of the statistical methods that will be used to analyse the trial data. This paper provides greater detail on the statistical analysis plan, including background and justification for the statistical methods chosen, in accordance with SPIRIT guidelines.

**Objective:**

To describe in detail the data management process and statistical methods that will be used to analyse the trial data.

**Methods:**

An overview of the trial design and primary and secondary research questions are provided. Sample size assumptions and calculations are explained, and randomisation and data management processes are described in detail. The planned statistical analyses for primary and secondary outcomes and sub-group analyses are specified along with the intended table layouts for presentation of the results.

**Conclusion:**

In accordance with best practice, all analyses outlined in the document are based on the aims of the study and have been pre-specified prior to the completion of data collection and outcome analyses.

**Trial registration:**

Australian New Zealand Clinical Trials Registry, ACTRN12616001372471. Registered on 3 August 2016.

## Background

The prevalence of type 2 diabetes (T2D) is rapidly increasing and is expected to reach close to 600 million worldwide by 2030 [1]. Close to 1.3 million Australians have been diagnosed with diabetes, with over 85% having T2D [2].

Early management and maintenance of glycaemic (blood glucose) levels through lifestyle modification and pharmacological treatments can reduce the likelihood of diabetes-related complications [3]. Glycated haemoglobin (HbA1c) is an index of average blood glucose level over the preceding 12 weeks and can be measured in mmol/mol or % [4]. HbA1c can be converted from one unit to the other using the relationship mmol/mol = 10.93 × HbA1c (%) – 23.5 [5]. Current guidelines base treatment intensification recommendations on HbA1c levels [6, 7]. The general HbA1c target in Australia is 53 mmol/mol (7%) [8]; however, the Australian Diabetes Society recommends that targets should also take into consideration factors such as age, duration of diabetes, and risk of hypoglycaemia [9]. Clinical care in general practice can help people with T2D achieve HbA1c targets [10] through adopting an evidence-based “treat-to-target” approach (step-wise treatment intensification through changes to lifestyle, medication doses, and/or prescription of additional medications). However, the majority of people with T2D have an HbA1c above their target level and treatment intensification is commonly delayed beyond clinical need [11]. One contributor to this may be that general practitioners (GP) and people with T2D lack an acceptable, feasible, simple, reliable, and effective method for identifying detailed day-to-day blood glucose patterns (glucose profiles) to guide decisions about treatment intensification.

Continuous glucose monitoring (CGM) is one method of identifying such glucose profiles and is measured in mmol/L. Retrospective CGM (r-CGM) involves the patient wearing a CGM sensor for a period of up to 2 weeks and then, usually in collaboration with their health professional, downloading the glucose data to identify day-to-day glucose profiles to guide treatment decisions. For many people with T2D glucose profiles tend to be stable over time and. Therefore. intermittent r-CGM measurements may be sufficient to guide clinical management. r-CGM can also provide detail about hypoglycaemia, hyperglycaemia, glycaemic variability (GV), i.e. the extent to which glucose fluctuates throughout the day as well as time spent in day-to-day glucose target range, all of which may be important to clinical and psychosocial outcomes for people with T2D [12, 13]. R-CGM thus offers the prospect of an advance in appropriate and personalised care for people with T2D [14].

General Practice Optimising Structured Monitoring to Improve Clinical Outcomes in Type 2 Diabetes (GP-OSMOTIC) is a stratified (by GP clinic) individually randomised controlled trial in general practice comparing the use of r-CGM (intervention) to usual care (control) in those with T2D whose HbA1c is above their individualised target level. Within each clinic, participants will be randomly allocated to either the intervention or control group. Full details of the trial method are described elsewhere [15], but are briefly outlined below before presenting the detailed description of the planned statistical methods.

### Primary objective

The primary objective was to assess whether the judicious use of intermittent retrospective continuous glucose monitoring (r-CGM) in people with T2D in primary care improves glycaemic control at 12 months as measured by HbA1c.

### Secondary objectives

Compared with the control arm, does the judicious use of intermittent retrospective continuous glucose monitoring (r-CGM) in people with T2D in primary care:

1. Improve the percentage of time spent in the target glucose range at 12 months?
2. Decrease diabetes-specific distress at 12 months?
3. Result in cost-effective care?
4. Decrease HbA1c at 6 months?

### Primary outcome

The primary outcome measure is the difference in mean HbA1c at 12 months between the intervention and control groups.

### Secondary outcomes

The secondary outcome measures are:

1. Difference in mean percent time in target (4–10 mmol/L) range at 12 months between the study groups (from data downloaded from the r-CGM device).
2. Difference in mean diabetes-specific distress at 12 months between the study groups as measured by the Problem Areas in Diabetes (PAID) scale [16].
3. Incremental cost per quality-adjusted life year (QALY) for the intervention relative to control for the trial period, as measured by the EuroQol 5 dimension 3 levels (EQ-5D-3 L) [17].
4. Difference in mean HbA1c (%) at 6 months between the intervention and control groups.

### Inclusion criteria

Eligible participants will be aged 18–80 years, active patients of the practice (defined as three or more visits to the practice in the last 2 years), have had T2D for at least 1 year with their most recent HbA1c (in the previous 1 month) ≥ 7 mmol/mol (0.5%) above their individualised target (see below) while on at least two non-insulin hypoglycaemic therapy and/or insulin (therapy stable for the last 4 months). Our general glycaemic target is set at 53 mmol/mol (7%) while patients with a history of severe hypoglycaemia (requiring assistance from a third person) or who report impaired awareness of hypoglycaemia (i.e. are unable or have reduced capacity to recognise the early signs and symptoms of hypoglycaemia, which may impede timely self-treatment) will have a target of 64 mmol/mol (8%). In the setting of this pragmatic trial we will allow GPs to indicate a personalised target for a participant if they feel that it should differ from the two pre-specified targets set out above.

Patient exclusion criteria will include: any debilitating medical condition (e.g. unstable cardiovascular disease (CVD), severe mental illness, end-stage cancer), an estimated glomerular filtration rate (eGFR) < 30 ml/min/1.73m^2^, proliferative retinopathy, pregnancy, lactating or planning pregnancy, unable to speak English/give informed consent, unwilling to use r-CGM or follow study protocol, allergy to adhesive tape, diagnosis of T2D within the past 12 months, and any condition that makes monitoring diabetes using HbA1c unreliable (e.g. haemoglobinopathy, iron deficiency anaemia).

### Randomisation

Participants will be stratified by clinic and randomised to either the intervention or control group using randomly permuted block sizes of 4 and 6. The randomisation process will be through REDCap© electronic data capture tools hosted at the University of Melbourne [18], using the application programming interface (API). This allows project information to be exported to a separate statistical computing package which generates allocation sequence tables allowing for random block sizes. These will then be imported back into REDCap© for use through the randomisation graphical user interface (GUI).

### Intervention

In brief, intervention group participants will be asked to wear the r-CGM device for a period of 2 weeks every 3 months, i.e. at baseline, 3, 6, 9, and 12 months, as well as having an HbA1c test at those times, and to attend a consultation with their GP (clinic assessment visit (CAV)) to discuss the r-CGM reports. This 3-monthly interval is in keeping with clinical practice guidelines [19]. Intervention participants will also attend a 60-min education session with the study registered nurse credentialed diabetes educator (RN-CDE) which will include instruction on how to wear the r-CGM device and how to interpret the glucose reports from the device to better understand their blood glucose and how this relates to their diabetes self-management and treatment options. The r-CGM device being used in the study is the Abbott FreeStyle Libre Pro® Flash Glucose Monitoring System.

Control group participants will wear the r-CGM device at baseline (blinded) and thereafter will be managed according to usual clinical care. The GP and patient will be prompted to undertake 3-monthly diabetes reviews in keeping with clinical practice guidelines about step-wise regular consideration of treatment intensification. Patients randomised to the control group will also attend an education session with a local CDE, funded by the study if required to ensure financial barriers do not exist. Control group participants will have an r-CGM sensing at 12 months, which will be used in collaboration with their GP in their management of diabetes after the final HbA1c blood measurement and all other trial outcomes have been collected.

### Outcome measures

The primary outcome, HbA1c, will be measured by venous blood test in an accredited laboratory. Time in the target range will be calculated as the percentage of time blood glucose levels remain between 4 and 10 mmol/L as measured by the r-CGM device. Diabetes-specific distress will be measured using the PAID scale [16]. This scale consists of 20 questions relating to negative emotions associated with diabetes, with five possible responses to each question: 0 = no problem, 1 = minor problem, 2 = moderate problem, 3 = somewhat serious problem, and 4 = serious problem. The 20 items are summed, and the total is multiplied by 1.25 so that total score ranges from 0 to 100. Higher scores indicate greater levels of diabetes-specific distress; a score of ≥ 40 indicates severe diabetes distress [20]. The PAID measure has high internal reliability and validity [16].

Results from the EQ-5D-3 L assessment at each measurement will be transformed into utility scores using Australian preference weights [21]. An average utility curve, which measures the mean quality of life trajectory for patients, will be derived by interpolating between baseline and the follow-up measurement points [22]. QALYs will then be estimated for both the intervention and the control group using the ‘area under the curve’ method [23]. As the economic evaluation will be performed within a 12-month period, discounting will not be applied.

### Statistical analysis

#### Sample size

The sample size is based on an individually randomised controlled trial without accounting for stratification by clinic. Clinical significance was considered to be a difference of at least 0.5% (7 mmol/mol) in mean HbA1c between the groups and is based on current guidelines which recommend intensification of therapy when HbA1c levels remain 0.5% (7 mmol/mol) above target [19]. The sample size was calculated using HbA1c in %. Using a significance level of 0.05, power of 0.8, clinically significant difference of 0.5%, and standard deviation of 1.3% for HbA1c [24], the required number of participants in each group is 108, a total of 216. This is equivalent to a difference in the mean HbA1c of 7 mmol/mol between the groups with a standard deviation of 14 mmol/mol [24]. Assuming a 20% attrition rate, the required sample size inflates to 270 (135 in each group). Allowing for 10% clinic attrition and assuming six participants per clinic, we require 50 clinics with six participants per clinic (150 in each group).

**Fig. 1:**
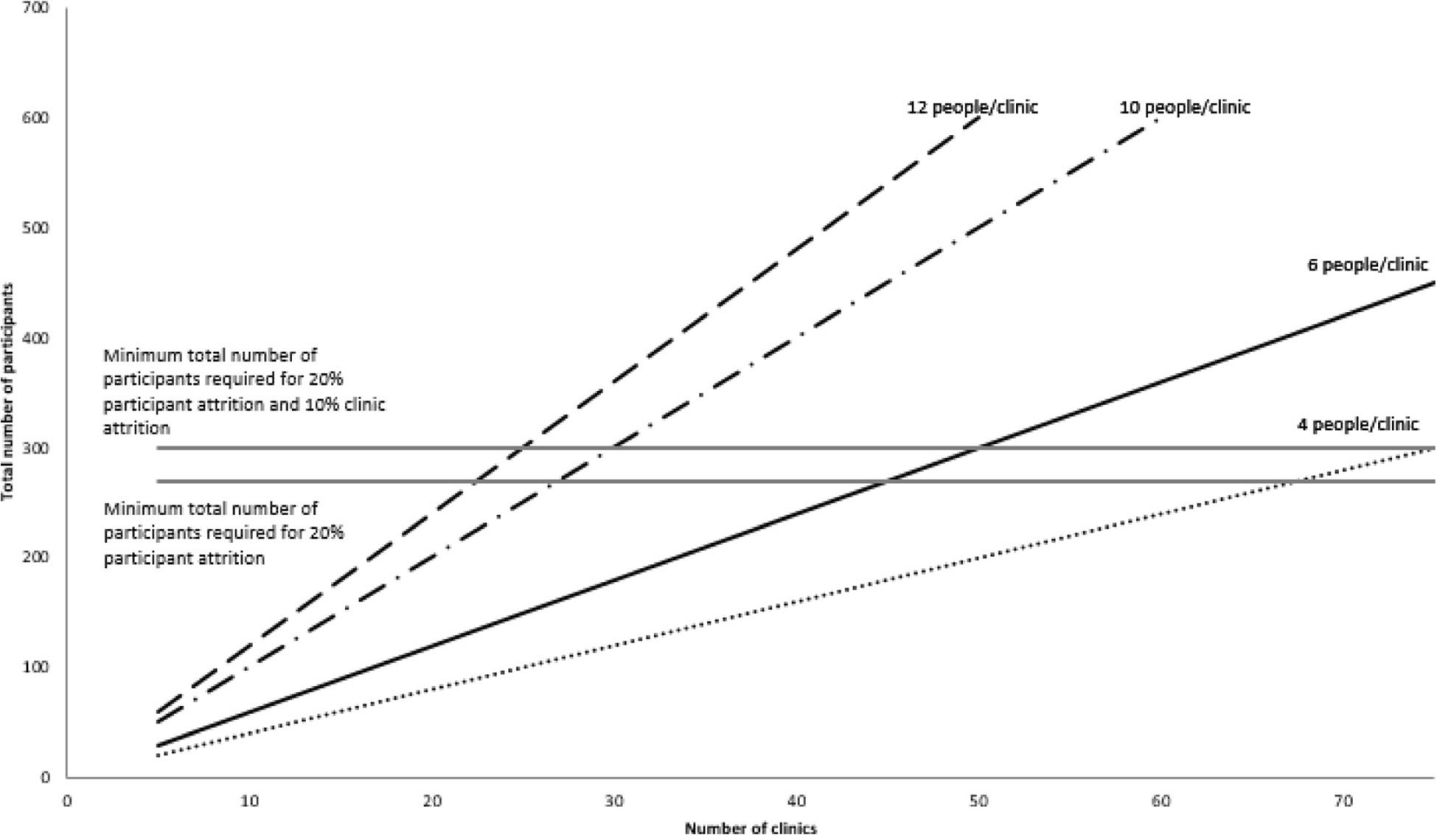
Total sample size versus number of clinics

Figure 1 shows the minimum number of clinics and participants per clinic required for 20% participant attrition and 10% clinic attrition. The figure shows that it is possible to recruit 300 participants in a variety of ways; for example, 25 clinics with 12 participants per clinic, 30 clinics with 10 participants per clinic, 50 clinics with six participants per clinic, and 75 clinics with four participants per clinic. Four participants per clinic was the minimum recommended to allow for estimation of the correlation in outcome measure between participants in the same group and clinic. From prior knowledge of recruitment patterns from the Stepping Up Study [24] it was decided to recruit 50 clinics with six participants per clinic.

#### Data collection and preparation

An in-house, web-based, purpose-built recruitment database will be used to document all practices approached to participate in the study. Once consented to the study, REDCap© will be used to store all clinic, GP, and practice nurse (PN) characteristics. All clinic, staff, and participant data will be collected at baseline and 12 months and entered into the database by research assistants using either a desktop computer or tablet. Data from CAVs and any technical issues or adverse events associated with the r-CGM device will be logged by research assistants in REDCap©.

HbA1c data will be collected 6-monthly from the same pathology laboratory for each patient and collated in a Microsoft Excel 2016 file. Participants will be encouraged to have their HbA1c levels collected at 3 and 9 months, but this will not be compulsory. The pathology data will be merged with the clinical patient data in STATA version 15.1 [25].

An in-house, web-based, purpose-built participant tracking database will be used to track changes in patient medication and the progress of patients throughout the study.

#### Trial profile

**Fig. 2:**
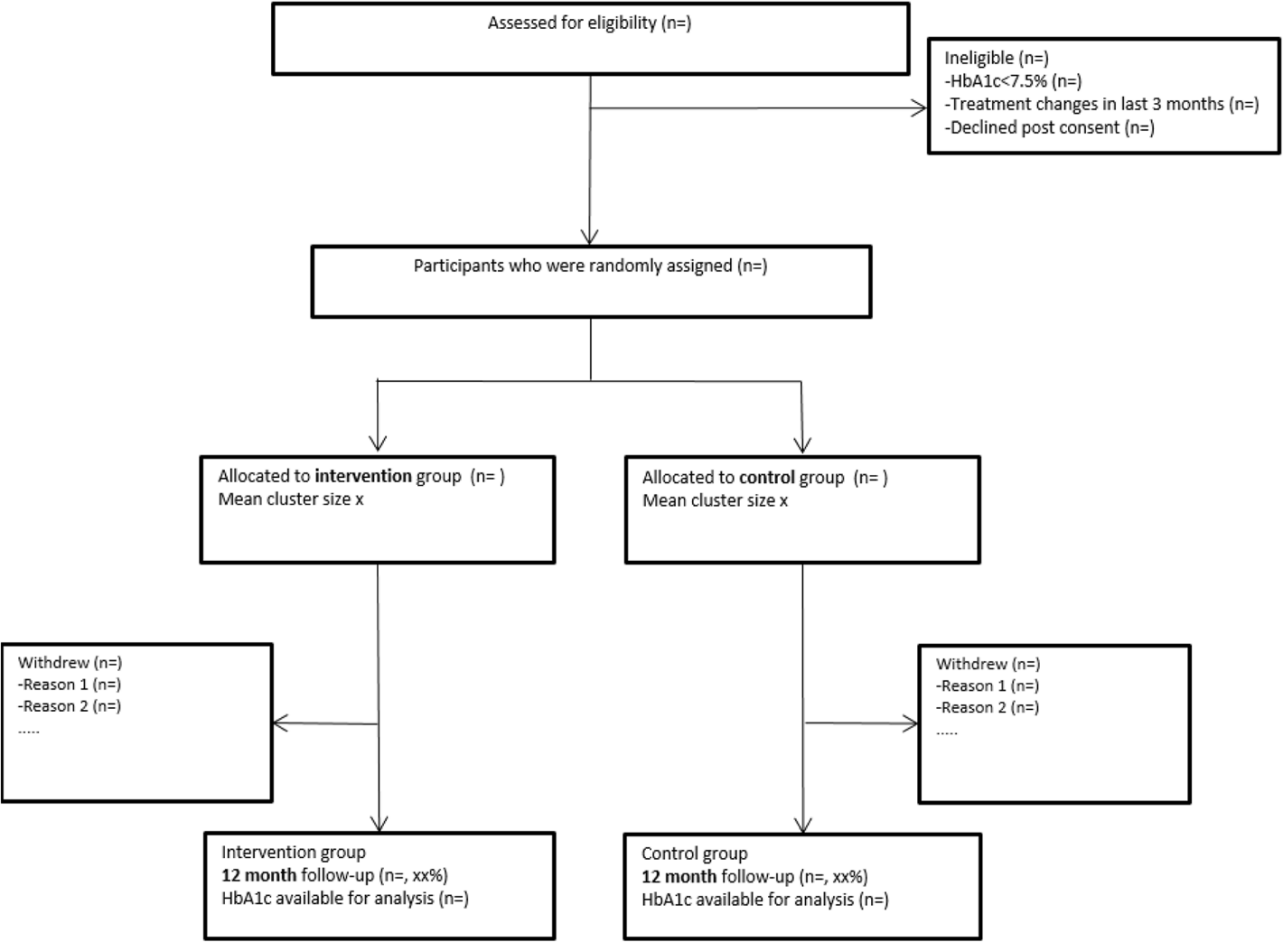
Study flow diagram to be completed for trial. HbA1c glycated haemoglobin

A study flow diagram (Fig. 2) will be used to summarise the progress of participants throughout the trial, from eligibility assessment to analysis of the primary outcome at 12 months [26].

#### Descriptive statistics

STATA version 15.1 (StataCorp, College Station, Texas) will be used for all analyses. Practice, GP, PN, and participant characteristics at baseline will be summarised (Tables 1 and 2). Continuous measures will be summarised using means and standard deviations or medians and interquartile ranges for skewed distributions. Categorical variables will be summarised using frequencies and percentages. Where applicable, the number of missing values will be specified and percentages for categorical variables will be based on the available data only.

**Table 1:** Baseline characteristics of practices, general practitioners (GPs), and practice nurses (PNs)

Characteristic	Measure	*n* (%)	Missing *n* (%)
Practices			
Practice billing			
Bulk billing	*n* (%)		
Private billing	*n* (%)		
Community health centre	*n* (%)		
Practice location			
Major city	*n* (%)		
Inner regional	*n* (%)		
Outer regional	*n* (%)		
Number of GPs per practice	Median (IQR)		
Number of PNs per practice	Median (IQR)		
GPs			
Age group (years)			
25–34	*n* (%)		
35–44	*n* (%)		
45–54	*n* (%)		
55–64	*n* (%)		
65 or over	*n* (%)		
Female	*n* (%)		
Working hours per week	Mean (SD)		
Years of experience	Mean (SD)		
PNs			
Age group (years)			
25–34	*n* (%)		
35–44	*n* (%)		
45–54	*n* (%)		
55–64	*n* (%)		
65 or over	*n* (%)		
Female	*n* (%)		
Working hours per week	Mean (SD)		
Years of experience	Mean (SD)		

*IQR* interquartile range, *SD* standard deviation

**Table 2:** Baseline characteristics of participants by study group

Participant characteristics		Intervention (*n*=)	Missing *n* (%)	Control (*n*=)	Missing *n* (%)
Age (years)	Mean (SD)				
Female	*n* (%)				
Country of birth					
Australia	*n* (%)				
Other	*n* (%)				
Highest level of education					
Primary or never attended	*n* (%)				
Secondary or trade/TAFE	*n* (%)				
University diploma/degree	*n* (%)				
Employed	*n* (%)				
IRSD (decile)	Median (IQR)				
Healthcare card holder	*n* (%)				
Diabetes duration (years)	Median (IQR)				
History of severe hypoglycaemia^a^	*n* (%)				
HbA1c					
mmol/mol	Median (IQR)				
%	Median (IQR)				
Individualised target over 7%	*n* (%)				
Diabetes distress (PAID)	Mean (SD)				
Severe diabetes distress (PAID ≥ 40)	*n* (%)				
Weight (kg)	Mean (SD)				
Blood pressure (mmHg)					
Systolic	Mean (SD)				
Diastolic	Mean (SD)				
Current medications					
Non-insulin hypoglycaemic agents					
Metformin	*n* (%)				
Sulphonylureas	*n* (%)				
DPP4i	*n* (%)				
GLP1	*n* (%)				
Other	*n* (%)				
Insulin	*n* (%)				
Number of hypoglycaemic agents					
1–2 agents	*n* (%)				
3 agents	*n* (%)				
4–5 agents	*n* (%)				

*GP* general practitioner, *HbA1c* glycated haemoglobin, *IQR* interquartile range, *IRSD* index of relative socio-economic disadvantage (calculated using patient postcode [33]), *PAID* problem area in diabetes, *PN* practice nurse, *SD* standard deviation, *TAFE* technical and further education

^a^ Hypoglycaemia requiring third party assistance

*Medicare is managed by the Department of Human Services and is Australia’s publicly funded healthcare system funding primary health care for Australian citizens and permanent residents

*The PBS is managed by the Department of Human Services and is a list of medicines available to be dispensed to patients at a government-subsidised price. The scheme is available for all Australian residents

*Public hospitals are funded by the state, territory and Australian governments, and managed by state and territory governments. Victorian Admitted Episodes Dataset (VAED) and the Victorian Emergency Minimum Dataset (VEMD) provide hospital costings for Victorian patients

#### Statistical modelling

##### Primary and secondary outcomes

Whilst our primary outcome is HbA1c at 12 months post-intervention, we will estimate the between-group difference in mean HbA1c at 6 and 12 months with the same linear mixed-effects model using restricted maximum likelihood estimation. As the data are longitudinal, HbA1c measured at baseline, 6 months, and 12 months will be included in the model as the dependent variable and study groups (intervention and control) and time of the pathology result (baseline, 6, and 12 months) will be collected as fixed effects. A two-way interaction term between study group and time will be included in the model to estimate the between-group difference in mean HbA1c at 6 and 12 months, but we will constrain the estimated baseline means to be equal. The model will include random intercepts for clinic (as individuals will be clustered within clinics) and individuals (as patient measures are repeated within individuals). An unstructured variance-covariance structure will be assumed for the random effects variables as correlations between measurements within individuals and correlations between measurements in participants from the same clinic are expected to be unique.

Age, index of relative socio-economic disadvantage (IRSD), and a history of severe hypoglycaemia are known to be at least moderately associated with HbA1c [9, 27]. In a secondary analysis, the outcome measure will be adjusted for these potential confounders. These measures will be included as fixed effects in the model.

An intention-to-treat (ITT) approach will be used where participants will be analysed according to the study group they were assigned, and all participants will be included in the analysis, consistent with mixed model analysis [28]. The estimated mean HbA1c levels at baseline, 6 months, and 12 months will be plotted for each study group with 95% confidence intervals.

The same statistical modelling approach described for HbA1c will be used for the secondary outcomes, percentage time in target and diabetes-specific distress at 12 months. Transformations for skewed outcome measures will be considered.

##### Economic evaluation

A within trial economic evaluation using participants’ Medicare costs, pharmaceutical benefit schedule (PBS) costs, hospitalisation costs, self-reported costs, diabetic outcomes (proportion with controlled diabetes, HbA1c ≤ 7 mmol/mol) and quality of life data will be performed using a decision analytic framework [29]. The economic model will construct costs and quality of life associated with the health states ‘controlled diabetes’, ‘uncontrolled diabetes’, and ‘death’. It will be constructed in STATA statistical software [25] based on the original trial data and will utilise linear and generalised linear modelling techniques to determine a cost per QALY gained. The analysis will be conducted from a health system and societal perspective. Costs and benefits will be bootstrapped. The distribution of costs and benefits will be simulated using a probabilistic analysis. The results of the economic modelling will be presented as the mean and 95% confidence interval (CI) of the incremental cost per QALY gained at trial conclusion for the r-CGM study group relative to the control group. Simulated cost-effectiveness will be presented for r-CGM relative to the control via a cost-effectiveness plane and a cost-effectiveness acceptability curve. Univariate and probabilistic sensitivity analyses will be performed to assess uncertainty. Estimates of projected implementation costs across Australia will be estimated.

#### Explanatory analysis

We will conduct two planned subgroup analyses for HbA1c at 6 and 12 months. In the first analysis, a two-way interaction term between history of severe hypoglycaemia (yes/no) and study group will be included in the primary analysis model to examine if there is a different intervention effect between those with a history of severe hypoglycaemia compared to those without. For the second subgroup analysis, a two-way interaction term between study group and type of HbA1c target (personalised vs general) will be added to the primary analysis model, to examine whether the intervention effect varies according to whether participants have a personalised HbA1c target that is different from the general target of 7% or not.

Results from the primary, secondary, and sub-analyses will be presented as shown in Tables 3, 4, 5, and 6. Estimates of the between-group difference for mean outcomes will be reported with their respective 95% confidence intervals and *p* values.

**Table 3:** Estimated HbA1c and between-group differences for intervention and control groups

	Estimated mean HbA1c (95% CI)		Difference in estimated mean HbA1c and general HbA1c target of 7%		Unadjusted		Adjusted*	
	Intervention group (*n*=)	Control group (*n*=)	Intervention group (*n*=)	Control group (*n*=)	Intervention vs. Control between-group differences (95% CI)	*P* Value	Intervention vs. Control between-group differences (95% CI)	*P* Value
Baseline								
6 months								
12 months								

*Adjusted for age, IRSD and history of severe hypoglycaemia

**Table 4:** Estimated percentage time in target, diabetes distress and between-group differences for intervention and control groups (secondary analyses)

	Estimated mean % time in target (95% CI)		Unadjusted		Adjusted*	
	Intervention group (*n*=)	Control group (*n*=)	Intervention vs. Control between-group differences (95% CI)	*P* Value	Intervention vs. Control between-group differences (95% CI)	*P* Value
Percentage time in target (%)						
Baseline						
12 months						
	Estimated mean diabetes distress (95% CI)		Unadjusted		Adjusted*	
	Intervention group (*n*=)	Control group (*n*=)	Intervention vs. Control between-group differences (95% CI)	*P* Value	Intervention vs. Control between-group differences (95% CI)	*P* Value
Diabetes-specific distress (PAID)						
Baseline						
12 months						

*Adjusted by age and IRSD

**Table 5:** Mean and standard deviation (SD) costs, mean and SD quality-adjusted life years (QALYs), mean differences and incremental cost-effectiveness ratios during the trial follow-up period for intervention vs control groups (secondary analyses)

	Intervention mean (SD)				Control mean (SD)		Mean difference (95% CI) per patient			*P* value
Costs										
Intervention (A)										
Hospital utilization (B)										
Medical benefits (C)										
Pharmaceuticals (D)										
Patient costs (E)										
Total cost health system (A + B + C + D)						(1)				
Total cost societal (A + B + C + D + E)						(2)				
QALYs						(3)				
Incremental cost effectiveness ratio (ICER)										
Health system perspective						(1)/(3)				
Societal perspective						(2)/(3)				

*CI* confidence interval

**Table 6:** Estimated glycated haemoglobin (HbA1c) and between-group differences by history of severe hypoglycaemia and personalised HbA1c target for intervention and control groups (sub-group analyses)

	Estimated mean HbA1c (95% CI)				Difference in estimated mean HbA1c and HbA1c target		Unadjusted		Adjusted^a^	
	Intervention group (*n*=)		Control group (*n*=)		Intervention group (*n*=)	Control group (*n*=)	Intervention vs. control between-group differences (95% CI)	*P* value	Intervention vs. control between-group differences (95% CI)	*P* value
Severe hypoglycaemia										
History of severe hypoglycaemia^b^										
Baseline										
6 months										
12 months										
No history of severe hypoglycaemia^c^										
Baseline										
6 months										
12 months										
Personalised HbA1c target										
Personalised target^d^										
Baseline										
6 months										
12 months										
No personalised target^e^										
Baseline										
6 months										
12 months										

*CI* confidence interval

^b^HbA1c target is 8%

^c^ HbA1c target is the general HbA1c target of 7%

^d^ HbA1c target based on average of personalised HbA1c targets

^e^ HbA1c target is the general HbA1c target of 7%

#### Complier average causal effect (CACE) analysis

A blinded review of compliance will be conducted by study investigators and the data management team prior to data analysis to determine whether a CACE analysis is required. If appropriate, CACE analysis will be performed on HbA1c at 12 months (primary outcome) to assess the size of the benefit of the intervention in those who comply with the intervention. Unlike a per-protocol analysis (PP), CACE analysis preserves randomisation when estimating the intervention effect [30]. This is achieved by comparing the mean HbA1c of ‘compliers’ in the intervention group (defined in Table 7) with a similar group of control participants who would have complied if they were offered the intervention. The outcome of the analysis is the CACE effect which represents the difference in mean HbA1c between compliers in the intervention group and their counterpart compliers in the control group.

**Table 7:** Definition of a complier for the complier average causal effect (CACE) analysis

The following four requirements must be met for a participant to be considered a complier:	
1. Participant attended the educational session at baseline with the study credentialed diabetes educator (CDE)	
2. General practitioner attended a face-to-face group education session or an education session with the study CDE or completed online training	
3. Participant wore a continuous glucose monitoring (CGM) sensor at baseline, 3 months, 6 months, and 9 months	
4. Participant attended clinic assessment visit (CAV) and discussed sensor trace at baseline, 3 months, 6 months, and 9 months	

The method assumes the same proportion of participants in the control group would have complied with the intervention if it was offered to them as those who did comply in the intervention group (A% in Table 8) [30]. Another important assumption is that mean HbA1c at 12 months is the same for non-compliers in both the intervention and control groups (x in Table 8) [30]. It is this assumption that allows the mean HbA1c of the (expected) compliers in the control group to be calculated (using the observed mean HbA1c in the control group). The CACE effect is then calculated as the difference in mean HbA1c between actual compliers in the intervention group and expected compliers control group. This will be reported with 95% confidence intervals.

**Table 8:** Complier average causal effect (CACE) analysis

Status	Intervention group		Control group	
	Proportion (%)	Mean HbA1c	Proportion (%)	Mean HbA1c
Complier	A%	y	A%	z
Non-complier	B%	x	B%	x
Overall		Observed mean HbA1c		Observed mean HbA1c

*HbA1c* glycated haemoglobin

#### Sensitivity analysis

The missing data patterns will be described and the drop-out rates between the two study groups will be compared. A sensitivity analysis will be performed on the primary analysis for HbA1c at 12 months to test the robustness of the missing data assumption using a pattern-mixture model. Under the mixed-effects model, missing data are assumed to be missing at random [28]. Under this assumption, the difference between the mean of the missing data and the mean of the observed data *δ* is assumed to be zero. In a pattern-mixture model, a range of plausible values for *δ* other than 0 will be considered, where positive values of *δ* would indicate that, on average, participants who have missing data have higher (worse) HbA1c than observed participants, and negative values of *δ* assume participants with missing data have lower (better) mean HbA1c than observed participants. Results for plausible values of *δ* will be examined to determine whether study conclusions change for departures from the missing at random assumption in the primary analysis.

## Discussion

The design effect is a multiplier applied to sample size calculations for an individually randomised trial to account for the sampling method, such as stratified or cluster randomisation. In this study, participants will be randomly allocated to study groups stratified by the clinic they attend. For stratified randomised trials the design effect is (1 – ICC), where the intraclass correlation coefficient (ICC) quantifies the correlation of outcomes within clinics. Applying this design effect to the sample size calculations will reduce the number of individuals required for the same power as an individually randomised controlled trial with no stratification when the ICC is greater than zero [31]. For this study, we chose the more conservative sample size that did not adjust for stratification by clinic, that is the ICC was assumed to be zero to avoid challenges associated with estimating the ICC.

Randomly permuted block sizes of 4 and 6 were chosen to minimise differences in the number of participants in each study group should recruitment stop abruptly in a clinic and to ensure adequate participants in each study group for estimation of clinic effects. Random effects were chosen to model the clinic effects as we assumed clinics involved were a random sample across Victoria. Furthermore, random-effects models can perform better than fixed-effects models in terms of power and efficiency when there are a small number of participants per clinic and there are treatment assignment imbalances within clinics [32]. Lastly, the mixed-effects model includes all data observed on the subjects and satisfies the intention-to-treat principle in the presence of missing outcome data, provided the missing at random assumption holds.

This analysis plan was written prior to completion of the trial data collection phase. Analyses are pre-specified, consistent with the study objectives, and not driven by the data. An outcomes paper based on this analysis plan will be available upon completion of data collection, which is anticipated in late 2018.

## Abbreviations

API: Application programming interface
CACE: Complier average causal effect
CAV: Clinic assessment visit
CDE: Credentialed diabetes educator
CGM: Continuous glucose monitoring
CVD: Cardiovascular disease
eGFR: Estimated glomerular filtration rate
EQ-5D-3 L: EuroQol 5 dimension 3 levels
GP: General practitioner
GP-OSMOTIC: General Practice Optimising Structured Monitoring to Improve Clinical Outcomes in Type 2 Diabetes
GUI: Graphical user interface
GV: Glycaemic variability
HbA1c: Glycated haemoglobin
ICC: Intraclass correlation coefficient
ICER: Incremental cost-effectiveness ratio
IRSD: Index of relative socio-economic disadvantage
ITT: Intention-to-treat
PAID: Problem Areas in Diabetes
PBS: Pharmaceutical benefit schedule
PN: Practice nurse
PP: Per-protocol analysis
QALY: Quality-adjusted life year
r-CGM: Retrospective continuous glucose monitoring
REDCap: Research electronic data capture
RN-CDE: Registered nurse credentialed diabetes educator
SD: Standard deviation
T2D: Type 2 diabetes
VAED: Victorian admitted episodes dataset
VEMD: Victorian emergency minimum dataset

## References

[1] Zimmet PZ, Magliano DJ, Herman WH, Shaw JE (2014). Diabetes: a 21st century challenge. Lancet Diabetes Endocrinol.

[2] NDSS. NDSS Diabetes Australia: all types of diabetes [Internet]. 2018. Available from: https://static.diabetesaustralia.com.au/s/fileassets/diabetes-australia/58f32006-8395-4d20-9097-13e1cfbdca70.pdf. [cited 2018 Aug 15]

[3] Holman RR, Paul SK, Bethel MA, Matthews DR, HAW N (2008). 10-year follow-up of intensive glucose control in type 2 diabetes. N Engl J Med.

[4] Sherwani SI, Khan HA, Ekhzaimy A, Masood A, Sakharkar MK (2016). Significance of HbA1c test in diagnosis and prognosis of diabetic patients. Biomark Insights.

[5] Jones GR, Barker G, Goodall I, Schneider H-G, Shephard MD, Twigg SM (2011). Change of HbA1c reporting to the new SI units | The Medical Journal of Australia. Med J Aust.

[6] McGuire H, Longson D, Adler A, Farmer A, Lewin I (2016). Guideline Development Group. Management of type 2 diabetes in adults: summary of updated NICE guidance. BMJ.

[7] Association AD (2016). Standards of medical care in diabetes—2016 abridged for primary care providers. Clin Diabetes.

[8] Gunton JE, Cheung NW, TME D, Zoungas S, Colagiuri S, Australian Diabetes Society (2014). A new blood glucose management algorithm for type 2 diabetes: a position statement of the Australian Diabetes Society. Med J Aust.

[9] Cheung NW, Conn JJ, d’Emden MC, Gunton JE, Jenkins AJ, Ross GP (2009). Position statement of the Australian Diabetes Society: individualisation of glycated haemoglobin targets for adults with diabetes mellitus. Med J Aust.

[10] Gæde P, Vedel P, Larsen N, GVH J, Parving H-H, Pedersen O (2003). Multifactorial intervention and cardiovascular disease in patients with type 2 diabetes. N Engl J Med.

[11] Gettings JV, O’Connor R, O’Doherty J, Hannigan A, Cullen W, Hickey L, et al. A snapshot of type two diabetes mellitus management in general practice prior to the introduction of diabetes cycle of care. Irish J Med Sci (1971). 2018; Available from: http://www.ncbi.nlm.nih.gov/pubmed/29417379. [cited 2018 Mar 12]. 10.1007/s11845-018-1754-9 29417379

[12] Barendse S, Singh H, Frier BM, Speight J (2012). The impact of hypoglycaemia on quality of life and related patient-reported outcomes in type 2 diabetes: a narrative review. Diabet Med.

[13] Nalysnyk L, Hernandez-Medina M, Krishnarajah G (2010). Glycaemic variability and complications in patients with diabetes mellitus: evidence from a systematic review of the literature. Diabetes, Obes Metab.

[14] Pickup JC (2015). Banting Memorial Lecture 2014 technology and diabetes care: appropriate and personalized. Diabet Med.

[15] Furler J, O’Neal DN, Speight J, Blackberry I, Manski-Nankervis J-A, Thuraisingam S (2018). GP-OSMOTIC trial protocol: an individually randomised controlled trial to determine the effect of retrospective continuous glucose monitoring (r-CGM) on HbA1c in adults with type 2 diabetes in general practice. BMJ Open.

[16] Polonsky WH, Anderson BJ, Lohrer PA, Welch G, Jacobson AM, Aponte JE (1995). Assessment of diabetes-related distress. Diabetes Care.

[17] EuroQol—a new facility for the measurement of health-related quality of life. Health Policy (New York). 1990;16(3):199–208 Available from: https://www.sciencedirect.com/science/article/pii/0168851090904219. [cited 2018 Mar 12]. 10.1016/0168-8510(90)90421-9 10109801

[18] Harris PA, Taylor R, Thielke R, Payne J, Gonzalez N, Conde JG (2009). Research electronic data capture (REDCap)—a metadata-driven methodology and workflow process for providing translational research informatics support. J Biomed Inform.

[19] General practice management of type 2 diabetes. 2016. Available from: https://static.diabetesaustralia.com.au/s/fileassets/diabetes-australia/5d3298b2-abf3-487e-9d5e-0558566fc242.pdf. [cited 2018 Mar 12]

[20] Snoek FJ, Kersch NYA, Eldrup E, Harman-Boehm I, Hermanns N, Kokoszka A (2011). Monitoring of Individual Needs in Diabetes (MIND): baseline data from the Cross-National Diabetes Attitudes, Wishes, and Needs (DAWN) MIND study. Diabetes Care.

[21] Viney R, Norman R, King MT, Cronin P, Street DJ, Knox S (2011). Time trade-off derived EQ-5D weights for australia. Value Heal.

[22] Billingham LJ, Abrams KR, Jones DR (1999). Methods for the analysis of quality-of-life and survival data in health technology assessment. Health Technol Assess.

[23] Drummond M. Methods for the economic evaluation of health care programmes [Internet]. 445 p. Available from: https://www.oupjapan.co.jp/en/node/8281?language=en. [cited 2018 Nov 27]

[24] Furler J, O’Neal D, Speight J, Manski-Nankervis J-A, Gorelik A, Holmes-Truscott E (2017). Supporting insulin initiation in type 2 diabetes in primary care: results of the Stepping Up pragmatic cluster randomised controlled clinical trial. BMJ.

[25] StataCorp LLC. Stata | Data analysis and statistical software [Internet]. 2018. Available from: https://www.stata.com/products/. [cited 2018 Mar 12]

[26] Zwarenstein M, Treweek S, Gagnier JJ, Altman DG, Tunis S, Haynes B (2008). Improving the reporting of pragmatic trials: an extension of the CONSORT statement. BMJ.

[27] Online R, Cross R, Bonney AD, Mayne DJ, Weston KM. Cross-sectional study of area-level disadvantage and glycaemic-related risk in community health service users in the Southern.IML Research (SIMLR) cohort Cross-sectional study of area-level disadvantage and glycaemic-related risk in community health service users in the Southern Cross-sectional study of area-level disadvantage and glycaemic-related risk in community health service users in the Southern.IML Research (SIMLR) cohort. IML Res cohort Aust Heal Rev. 2017;1–7. Available from: http://ro.uow.edu.au/cgi/viewcontent.cgi?article=6003&context=smhpapers. [cited 2018 Mar 14]

[28] White IR, Carpenter J, Horton NJ (2012). Including all individuals is not enough: lessons for intention-to-treat analysis. Clin Trials J Soc Clin Trials.

[29] Briggs AH, Claxton K, Sculpher MJ. Decision modelling for health economic evaluation. New York: Oxford University Press Inc.; 2006. p. 237.

[30] Dunn G, Maracy M, Dowrick C, Ayuso-Mateos JL, Dalgard OS, Page H (2003). Estimating psychological treatment effects from a randomised controlled trial with both non-compliance and loss to follow-up. Br J Psychiatry.

[31] Vierron E, Giraudeau B (2009). Design effect in multicenter studies: gain or loss of power?. BMC Med Res Methodol.

[32] Kahan BC, Morris TP (2013). Analysis of multicentre trials with continuous outcomes: when and how should we account for centre effects?. Stat Med.

[33] Australian Bureau of Statisitcs. IRSD. Available from: http://www.abs.gov.au/ausstats/abs@.nsf/Lookup/2033.0.55.001main+features100052011. [cited 2018 Mar 12]

